# Genetic Structure of Modern Durum Wheat Cultivars and Mediterranean Landraces Matches with Their Agronomic Performance

**DOI:** 10.1371/journal.pone.0160983

**Published:** 2016-08-11

**Authors:** Jose Miguel Soriano, Dolors Villegas, Maria Jose Aranzana, Luis F. García del Moral, Conxita Royo

**Affiliations:** 1 Field Crops Programme, Institut de Recerca i Tecnología Agroalimentaries, Lleida, Spain; 2 Plant and Animal Genomics Programme, Centre de Recerca en Agrigenómica, Bellaterra, Barcelona, Spain; 3 Department of Plant Physiology, Institute of Biotechnology, University of Granada, Granada, Spain; Università Politecnica delle Marche, ITALY

## Abstract

A collection of 172 durum wheat landraces from 21 Mediterranean countries and 20 modern cultivars were phenotyped in 6 environments for 14 traits including phenology, biomass, yield and yield components. The genetic structure of the collection was ascertained with 44 simple sequence repeat markers that identified 448 alleles, 226 of them with a frequency lower than 5%, and 10 alleles per locus on average. In the modern cultivars all the alleles were fixed in 59% of the markers. Total genetic diversity was *H*_*T*_ = 0.7080 and the genetic differentiation value was *G*_*ST*_ = 0.1730. STRUCTURE software allocated 90.1% of the accessions in five subpopulations, one including all modern cultivars, and the four containing landrace related to their geographic origin: eastern Mediterranean, eastern Balkans and Turkey, western Balkans and Egypt, and western Mediterranean. Mean yield of subpopulations ranged from 2.6 t ha^-1^ for the western Balkan and Egyptian landraces to 4.0 t ha^-1^ for modern cultivars, with the remaining three subpopulations showing similar values of 3.1 t ha^-1^. Modern cultivars had the highest number of grains m^-2^ and harvest index, and the shortest cycle length. The diversity was lowest in modern cultivars (*H*_*T*_ = 0.4835) and highest in landraces from the western Balkans and Egypt (*H*_*T*_ = 0.6979). Genetic diversity and AMOVA indicated that variability between subpopulations was much lower (17%) than variability within them (83%), though all subpopulations had similar biomass values in all growth stages. A dendrogram based on simple sequence repeat data matched with the clusters obtained by STRUCTURE, improving this classification for some accessions that have a large admixture. landraces included in the subpopulation from the eastern Balkans and Turkey were separated into two branches in the dendrogram drawn with phenotypic data, suggesting a different origin for the landraces collected in Serbia and Macedonia. The current study shows a reliable relationship between genetic and phenotypic population structures, and the connection of both with the geographic origin of the landraces.

## Introduction

Durum wheat (*Triticum turgidum* L. var. *durum*) is a traditional Mediterranean crop. It originated in the Fertile Crescent (10,000 BP) and spread over the northern side of the Mediterranean, reaching the Iberian Peninsula in about 7000 BP [1] from both Italy and North Africa [2]. During this migration, natural and human selection processes resulted in the development of local landraces that were widely cultivated until the middle of the 20th century. From then, as a consequence of the Green Revolution, the cultivation of local landraces was progressively abandoned and they were replaced by the improved, more productive and genetically uniform semi-dwarf cultivars. The plant height (PH), general lateness and low harvest index (HI) of landraces have restricted their current cultivation to a few marginal areas or to the framework of organic farming, discouraging wheat breeding programmes from evaluating and using them extensively as parents in crossings.

Nevertheless, scientists are convinced that local landraces may provide new alleles for the improvement of commercially valuable traits [3]. Introgression of these alleles into modern cultivars can be very useful, especially in breeding for suboptimal environments. In the Mediterranean Basin durum wheat is mostly cultivated in rainfed environments, in areas where the amount and occurrence of rains fluctuate drastically between years and between locations within a year, resulting in major yield variations. Therefore, improving yield under water-limited conditions is one of the major challenges for wheat production, particularly in the current scenario of climate change. Mediterranean durum wheat landraces represent a particularly important group of genetic resources that are useful for breeding because of a number of suitable characteristics: good adaptation to the regions where they are grown, huge genetic diversity [4], a documented resilience to abiotic stresses [5], and resistance to pests and diseases [6]. An increase in the available genetic variation through the use of landraces in breeding programmes therefore seems possible in terms of adaptation to harsh environments and end-product quality, given the high level of polymorphism found between and within landraces for traits of commercial importance [3, 7–9].

Knowledge of genetic diversity is essential for understanding the relationships between cultivars, facilitating their classification and characterization with the aim of defining new selection strategies and crosses in breeding programmes. Although several markers have been used in the last few decades for genetic studies [10], molecular markers based on microsatellite repeats (SSR—simple sequence repeat) have been the ones most used in wheat during the last few years because of their wide distribution in the genome, their codominancy, their high polymorphism and reproducibility, and their simplicity of analysis. A number of studies have confirmed SSR markers as an efficient tool for evaluating the genetic diversity of wheat germplasm collections and assessing subpopulation structure [11–22].

However, fine phenotyping is a major challenge for the improvement of cultivars, creating a bottleneck in the breeding process, especially for the quantitative traits that are the major determinants of abiotic stress resistance. Therefore, accurate phenotyping is essential to minimize the experimental errors due to uncontrolled environmental and experimental variability, and to reduce the genotype-phenotype gap.

To date, few studies have examined the relationship between genetic population structure and agronomic performance in wheat. Previous works [23, 24] using collections of 30 bread wheat and 24 durum wheat accessions, respectively, revealed little correlation between phenotypic traits and genetic diversity based on molecular markers. More recently [14], using a set of 191 elite durum wheat genotypes representative of the genetic diversity present in the Mediterranean durums, the authors suggested that genotypic proximity corresponded to agronomic performance in only a few cases. Good correlation between phenotypic and molecular structures was found for accessions related to the CIMMYT hallmark founder ‘Altar 84’, for ICARDA accessions adapted to dryland areas, and for the reduced set of landraces used in that study.

The aims of this study were: 1) to determine the diversity existing in a durum wheat collection of 20 modern cultivars and 172 landraces representative of the variability existing in the species within the Mediterranean Basin, 2) to ascertain the genetic structure of the collection, and 3) to study the relationship between the genetic and geographic structures and the cluster based on the agronomic performance of the collection across six environments.

## Materials and Methods

### Plant material

The plant material included a collection of 172 durum wheat landraces and old varieties from 21 Mediterranean countries, and 20 modern cultivars used as reference, which were previously selected by [25] (S1 Table). Landraces were selected from a larger collection comprising 231 accessions of different origin based on genetic variability determined by 33 SSR markers in order to represent the genetic diversity of ancient local durums from the Mediterranean Basin ([4]. Landraces provided by public gene banks (*Centro de Recursos Fitogenéticos* INIA-Spain, ICARDA Germplasm Bank and USDA Germplasm Bank) were bulk purified to select the dominant type (usually with a frequency above 80% of the bulk) and the seed was increased in plots planted in the same field in the years before each experiment to ensure a common origin for seeds of all lines. The modern set included Spanish, Italian and French cultivars, as well as the US desert durum cultivar ‘Ocotillo’ (S1 Table).

### Molecular profiling

DNA isolation was performed from leaf samples following the method reported by Doyle and Doyle [26]. Forty-four SSR markers widely distributed along the genome and amplifying polymorphic alleles in previous studies [27–29] were chosen. SSR primer sequence and amplification conditions were obtained from the GrainGenes database (http://wheat.pw.usda.gov). The forward primer of each marker was 5’-labelled with a fluorescence tag and allele sizes were determined using an ABI Prism 3130xl Genetic Analyser with the GeneMapper software version 4.0 (Applied Biosystems).

### Field experiments

Experiments were carried out in the 2007, 2008 and 2009 harvesting seasons in Lleida (41°40’N, 0°20’E, 260 m.a.s.l), northeastern Spain, and Granada (37°15’N, 3°46’W, 680 m.a.s.l), southern Spain. Soil analyses were performed before sowing. Experiments were carried out in a non-replicated modified augmented design with three replicated checks (the cultivars ‘Claudio’, ‘Simeto’ and ‘Vitron’) and plots of 6 m^2^ (8 rows, 5 m long with a 0.15 m spacing). Sowing density was adjusted to 250 viable seeds m^-2^. Meteorological data (S2 Table) were recorded by weather stations placed in the experimental fields. Experiments were conducted under rainfed conditions, but the lack of rain after sowing in 2007 made irrigation necessary to allow seed germination. Weeds and diseases were controlled according to the standard cultural practices of each site.

Zadoks growth stages (GS) [30] 21 (beginning of tillering), 33 (mid-jointing), 45 (booting), 55 (heading), 65 (anthesis), and 87 (physiological maturity) were determined in each plot. Samples of the plants in a 0.5-m-long row were pulled up in a central row of each plot at GS21, GS33 and GS65, and a 1-m-long row from a central row of each plot was taken at GS87. In the laboratory, the number of plants, stems and spikes in each sample were counted, and the aerial portion was weighed after being oven-dried at 70°C for 48 h. Crop dry weight (CDW g m^-2^) was then calculated for each sample as the product of average dry weight per plant and the number of plants m^-2^, as described by Royo et al. [25]. The number of spikes per square metre (NSm^2^) and the number of grains per square metre (NGm^2^) were measured at GS87. HI was calculated as the ratio between grain and aerial biomass weight on a whole sample basis. PH was measured at anthesis in ten main stems per plot from the tillering node to the top of the spike, excluding the awns. Plots were mechanically harvested at ripening and grain yield (t ha^-1^) was expressed on the basis of 12% moisture. Thousand kernel weight (TKW) was estimated as the mean weight of three sets of 100 g per plot.

### Data analysis

The following variables were estimated from the SSR marker data using the GenAlEx software version 6.502 [31]: number of alleles per locus (*Na*); expected heterozygosity (*He* = 1 − *Σ p*_*i*_^*2*^, where *p*_*i*_ is the frequency of the *i*_th_ allele) [32]; observed heterozygosity (*Ho*, calculated as the number of heterozygous genotypes divided by the total number of genotypes); and fixation index (*F = 1–Ho/He*) [33] (Table 1). Putative population structure was estimated using the STRUCTURE software version 2.1 [34], adopting an admixture model and correlated alleles, with burn-in and MCMC 10,000 and 100,000 cycles, respectively. A continuous series of *K* were tested from 2 to 11 in seven independent runs. The most likely number of subpopulations was calculated according to Evanno’s test (*ΔK*) [35]. Genetic diversity was estimated with the total diversity (*H*_*T*_) [32] using POPGENE version 1.32 [36]. The coefficient of genetic differentiation, i.e. the proportion of total variation that is distributed between populations (*G*_*ST*_), was calculated as *G*_*ST*_ = *D*_*ST*_/*H*_*T*_ w, where *D*_*ST*_ is the genetic diversity between populations calculated as *D*_*ST*_ = *H*_*T*_*—H*_*S*_, where *H*_*S*_ is the mean genetic diversity within populations. Genetic distances between groups were calculated according to Nei’s genetic distance [37], and cluster analysis of the different populations was carried out using the unweighted pair-group method (UPGMA) with DARWin software version 6.0.11 [38]. Analysis of molecular variance (AMOVA) was used to assess the variance between and within populations from different geographical origins with the GenAlEx software version 6.502 [31].

**Table 1:** SSR loci.

		All genotypes					Subpopulation 1					Subpopulation 2					Subpopulation 3					Subpopulation 4					Subpopulation 5				
Locus	Chr	N	Na	Ho	He	F	N	Na	Ho	He	F	N	Na	Ho	He	F	N	Na	Ho	He	F	N	Na	Ho	He	F	N	Na	Ho	He	F
**Barc158**	1A	157	4	0.01	0.33	0.96	54	3	0.02	0.16	0.88	20	2	0.00	0.50	1.00	18	2	0.00	0.50	1.00	21	2	0.05	0.13	0.64	27	2	0.00	0.07	1.00
**Barc263**	1A	178	6	0.08	0.61	0.87	70	5	0.07	0.44	0.84	20	2	0.00	0.10	1.00	19	4	0.00	0.66	1.00	19	3	0.05	0.23	0.78	33	4	0.15	0.63	0.76
**cfa2135**	1A	188	4	0.02	0.43	0.96	71	2	0.00	0.49	1.00	20	2	0.00	0.42	1.00	21	3	0.00	0.25	1.00	20	2	0.00	0.10	1.00	37	3	0.05	0.31	0.83
**Barc008**	1B	165	13	0.05	0.86	0.94	60	9	0.03	0.80	0.96	18	5	0.00	0.72	1.00	20	4	0.10	0.37	0.73	19	5	0.11	0.67	0.84	32	10	0.03	0.86	0.96
**Barc080**	1B	173	5	0.08	0.30	0.75	63	2	0.06	0.15	0.57	20	2	0.00	0.10	1.00	19	4	0.21	0.49	0.57	19	3	0.16	0.47	0.66	36	2	0.03	0.13	0.79
**gwm018**	1B	185	5	0.05	0.72	0.93	73	4	0.08	0.60	0.86	20	3	0.00	0.27	1.00	19	3	0.00	0.53	1.00	21	4	0.00	0.65	1.00	33	5	0.06	0.78	0.92
**wmc453**	2A	173	18	0.25	0.91	0.73	60	15	0.25	0.86	0.71	20	5	0.00	0.66	1.00	20	6	0.05	0.71	0.93	20	9	0.25	0.82	0.70	36	13	0.42	0.88	0.53
**Barc55A**	2B	190	6	0.07	0.76	0.90	73	5	0.07	0.70	0.90	20	6	0.05	0.69	0.93	21	3	0.00	0.38	1.00	20	3	0.05	0.30	0.84	37	6	0.08	0.69	0.88
**Barc55B**	2B	191	8	0.16	0.76	0.79	73	8	0.11	0.71	0.85	19	6	0.47	0.76	0.37	21	5	0.14	0.44	0.68	21	4	0.10	0.26	0.63	38	7	0.05	0.71	0.93
**wmc175**	2B	190	16	0.10	0.85	0.88	72	12	0.06	0.82	0.93	20	5	0.05	0.56	0.91	21	9	0.29	0.79	0.64	20	2	0.00	0.18	1.00	38	9	0.13	0.76	0.83
**wmc25**	2B	191	10	0.05	0.70	0.93	73	6	0.04	0.65	0.94	20	3	0.00	0.52	1.00	20	5	0.00	0.62	1.00	21	5	0.10	0.70	0.86	38	8	0.05	0.77	0.93
**cfa2134**	3A	180	21	0.06	0.93	0.93	69	14	0.06	0.89	0.94	17	7	0.00	0.76	1.00	21	7	0.05	0.77	0.94	20	7	0.05	0.72	0.93	36	15	0.06	0.90	0.94
**gwm155**	3A	166	11	0.13	0.86	0.85	62	10	0.15	0.77	0.81	14	3	0.00	0.26	1.00	18	6	0.17	0.71	0.77	21	8	0.14	0.80	0.82	33	9	0.06	0.75	0.92
**wmc532**	3A	182	13	0.04	0.71	0.95	72	9	0.03	0.41	0.93	20	3	0.00	0.41	1.00	20	7	0.10	0.78	0.87	21	6	0.00	0.47	1.00	32	8	0.06	0.75	0.92
**Barc1077**	3B	189	8	0.05	0.46	0.90	71	8	0.06	0.57	0.90	20	2	0.00	0.26	1.00	21	5	0.00	0.40	1.00	21	3	0.00	0.38	1.00	37	7	0.14	0.43	0.69
**gwm247**	3B	167	15	0.17	0.86	0.80	58	10	0.19	0.84	0.77	20	7	0.25	0.81	0.69	19	6	0.05	0.75	0.93	20	7	0.10	0.73	0.86	35	10	0.23	0.84	0.73
**gwm493**	3B	164	12	0.06	0.71	0.91	62	10	0.08	0.56	0.86	20	2	0.00	0.32	1.00	18	4	0.00	0.67	1.00	20	3	0.00	0.19	1.00	32	9	0.13	0.83	0.85
**gwm566**	3B	189	11	0.15	0.81	0.81	72	8	0.15	0.68	0.78	20	5	0.10	0.60	0.83	20	4	0.10	0.57	0.82	21	5	0.24	0.63	0.62	37	10	0.16	0.85	0.81
**wmc420**	4A	187	9	0.03	0.64	0.96	71	6	0.01	0.68	0.98	20	1	0.00	0.00	-	21	5	0.05	0.46	0.90	19	4	0.05	0.43	0.88	37	7	0.03	0.73	0.96
**wmc468**	4A	192	7	0.08	0.58	0.87	73	6	0.08	0.65	0.87	20	5	0.10	0.62	0.84	21	5	0.00	0.51	1.00	21	5	0.10	0.46	0.79	38	4	0.05	0.48	0.89
**wms601**	4A	188	4	0.95	0.58	-0.65	71	4	0.92	0.53	-0.72	20	3	1.00	0.52	-0.91	21	3	0.95	0.58	-0.63	20	2	0.90	0.50	-0.82	37	4	1.00	0.67	-0.50
**wms610**	4A	192	14	0.20	0.88	0.78	73	11	0.18	0.84	0.79	20	5	0.05	0.61	0.92	21	8	0.14	0.77	0.81	21	8	0.33	0.84	0.60	38	11	0.29	0.87	0.67
**Barc1045**	4B	191	6	0.09	0.61	0.85	72	5	0.08	0.49	0.83	20	1	0.00	0.00	-	21	4	0.14	0.22	0.34	21	2	0.05	0.39	0.88	38	4	0.08	0.54	0.85
**cfa2091**	4B	192	6	0.88	0.62	-0.40	73	5	0.88	0.55	-0.60	20	3	0.95	0.57	-0.68	21	5	0.86	0.62	-0.38	21	2	0.90	0.50	-0.83	38	4	0.87	0.67	-0.30
**wmc710**	4B	173	19	0.14	0.92	0.85	70	14	0.14	0.89	0.84	18	7	0.00	0.73	1.00	20	8	0.10	0.75	0.87	20	10	0.10	0.79	0.87	27	15	0.22	0.87	0.75
**Barc155**	5A	192	9	0.10	0.79	0.87	73	9	0.10	0.72	0.87	20	5	0.05	0.69	0.93	21	7	0.10	0.79	0.88	21	6	0.24	0.71	0.67	38	8	0.11	0.66	0.84
**gwm156**	5A	173	10	0.24	0.87	0.73	60	10	0.18	0.79	0.77	18	4	0.00	0.59	1.00	20	8	0.30	0.67	0.55	21	8	0.38	0.74	0.49	36	9	0.31	0.82	0.63
**gwm205**	5A	188	8	0.07	0.40	0.83	73	4	0.04	0.36	0.89	20	2	0.00	0.10	1.00	20	4	0.10	0.37	0.73	21	3	0.00	0.18	1.00	35	8	0.23	0.61	0.63
**wmc150b**	5A	186	9	0.04	0.85	0.95	69	8	0.03	0.79	0.96	20	6	0.00	0.76	1.00	21	7	0.14	0.77	0.81	20	4	0.00	0.59	1.00	38	7	0.03	0.80	0.97
**Barc1032**	5B	189	6	0.05	0.26	0.82	71	6	0.04	0.20	0.79	20	1	0.00	0.00	-	21	4	0.05	0.33	0.86	20	3	0.05	0.10	0.48	38	4	0.05	0.39	0.87
**Barc140**	5B	192	5	0.09	0.57	0.83	73	3	0.07	0.51	0.87	20	2	0.00	0.42	1.00	21	3	0.24	0.59	0.60	21	4	0.10	0.68	0.86	38	4	0.11	0.55	0.81
**wms67**	5B	189	5	0.02	0.45	0.96	71	4	0.01	0.43	0.97	20	2	0.00	0.10	1.00	21	2	0.05	0.05	-0.02	21	1	0.00	0.00	-	37	4	0.00	0.46	1.00
**Barc003**	6A	191	10	0.16	0.53	0.70	73	7	0.18	0.62	0.71	20	3	0.10	0.26	0.62	21	4	0.24	0.29	0.19	20	2	0.05	0.05	-0.03	38	9	0.13	0.64	0.79
**Barc107**	6A	169	10	0.06	0.81	0.93	62	8	0.11	0.83	0.86	18	5	0.06	0.30	0.81	20	4	0.00	0.70	1.00	19	5	0.00	0.61	1.00	33	6	0.03	0.79	0.96
**Barc354**	6B	188	18	0.03	0.92	0.97	70	15	0.01	0.90	0.98	20	6	0.00	0.82	1.00	21	6	0.00	0.67	1.00	21	10	0.10	0.86	0.89	37	13	0.05	0.89	0.94
**gwm494**	6B	178	11	0.03	0.84	0.96	67	9	0.03	0.76	0.96	19	8	0.05	0.76	0.93	18	7	0.00	0.82	1.00	20	7	0.05	0.73	0.93	36	10	0.06	0.87	0.94
**wmc486**	6B	123	6	0.01	0.64	0.99	53	4	0.00	0.58	1.00	19	3	0.00	0.50	1.00	15	3	0.00	0.42	1.00	8	3	0.13	0.59	0.79	12	5	0.00	0.69	1.00
**Barc151**	7A	183	15	0.06	0.90	0.93	68	8	0.04	0.82	0.95	20	6	0.00	0.73	1.00	21	8	0.10	0.83	0.89	21	8	0.10	0.80	0.88	36	12	0.06	0.87	0.94
**gwm282**	7A	190	12	0.10	0.83	0.88	73	11	0.11	0.72	0.85	19	3	0.00	0.42	1.00	21	5	0.10	0.63	0.85	21	5	0.05	0.67	0.93	37	8	0.16	0.81	0.80
**cfa2106**	7B	191	10	0.95	0.68	-0.40	72	9	0.96	0.64	-0.51	20	4	0.85	0.54	-0.59	21	5	0.95	0.60	-0.58	21	5	1.00	0.68	-0.46	38	8	0.95	0.76	-0.25
**gwm537**	7B	175	15	0.07	0.88	0.92	69	11	0.10	0.88	0.88	11	5	0.00	0.74	1.00	20	8	0.05	0.80	0.94	20	5	0.00	0.70	1.00	37	12	0.05	0.88	0.94
**wmc517**	7B	189	11	0.04	0.82	0.95	71	7	0.01	0.76	0.98	20	4	0.00	0.70	1.00	20	6	0.10	0.68	0.85	21	3	0.05	0.47	0.90	38	10	0.05	0.81	0.94
**wms333**	7B	187	14	0.14	0.87	0.84	69	11	0.16	0.84	0.81	20	6	0.00	0.60	1.00	21	9	0.19	0.78	0.75	21	7	0.19	0.56	0.66	37	8	0.14	0.80	0.83
**wms537**	7B	187	13	0.05	0.85	0.94	71	11	0.03	0.83	0.97	20	5	0.10	0.54	0.81	20	5	0.05	0.51	0.90	21	2	0.00	0.44	1.00	38	11	0.08	0.84	0.91
**Mean**		182	10	0.14	0.71	0.79	69	8	0.14	0.65	0.77	19	4	0.10	0.48	0.82	20	5	0.14	0.58	0.73	20	5	0.14	0.51	0.71	35	8	0.16	0.70	0.77

SSR loci analysed for the whole set of genotypes and in the five subpopulations (SP) estimated by STRUCTURE analysis. Locus, chromosome (Chr), number of genotypes (N), number of alleles (Na), observed heterozygosity (Ho), expected heterozygosity (He) and fixation index (F). SP1, western Mediterranean; SP2, modern cultivars; SP3, eastern Balkans & Turkey; SP4, eastern Mediterranean; SP5, western Balkans & Egypt.

Phenotypic data were fitted to a linear mixed model considering the check cultivars, the row and column number and accessions as fixed in the model for each environment. Restricted maximum likelihood was used to estimate the variance components and to produce the best linear unbiased estimates (BLUEs) for the phenotypic data of each accession within each environment using Genstat software version 17 (VSN International). Correlation analyses between traits were calculated using Genstat software version 17 using the mean values of the BLUEs. Analyses of variance (ANOVA) were performed for each phenotypic trait, considering the genotype (G) and the environment (E) (combination of year and location) as the sources of variation using the SAS Enterprise Guide software version 4.2 (SAS Institute Inc, Cary, NC, USA).

Diversity analysis between durum wheat accessions was conducted using both molecular and phenotypic data. Genetic distances between durum wheat accessions were determined using the simple matching coefficient [39] and phenotypic relationships were determined from the Euclidean distances calculated with the standardized mean phenotypic data across environments implemented in the DARWin software version 6.0.11 [38]. Un-rooted trees were calculated using the neighbour-joining clustering method [40].

## Results

### Molecular analyses

**Fig 1:**
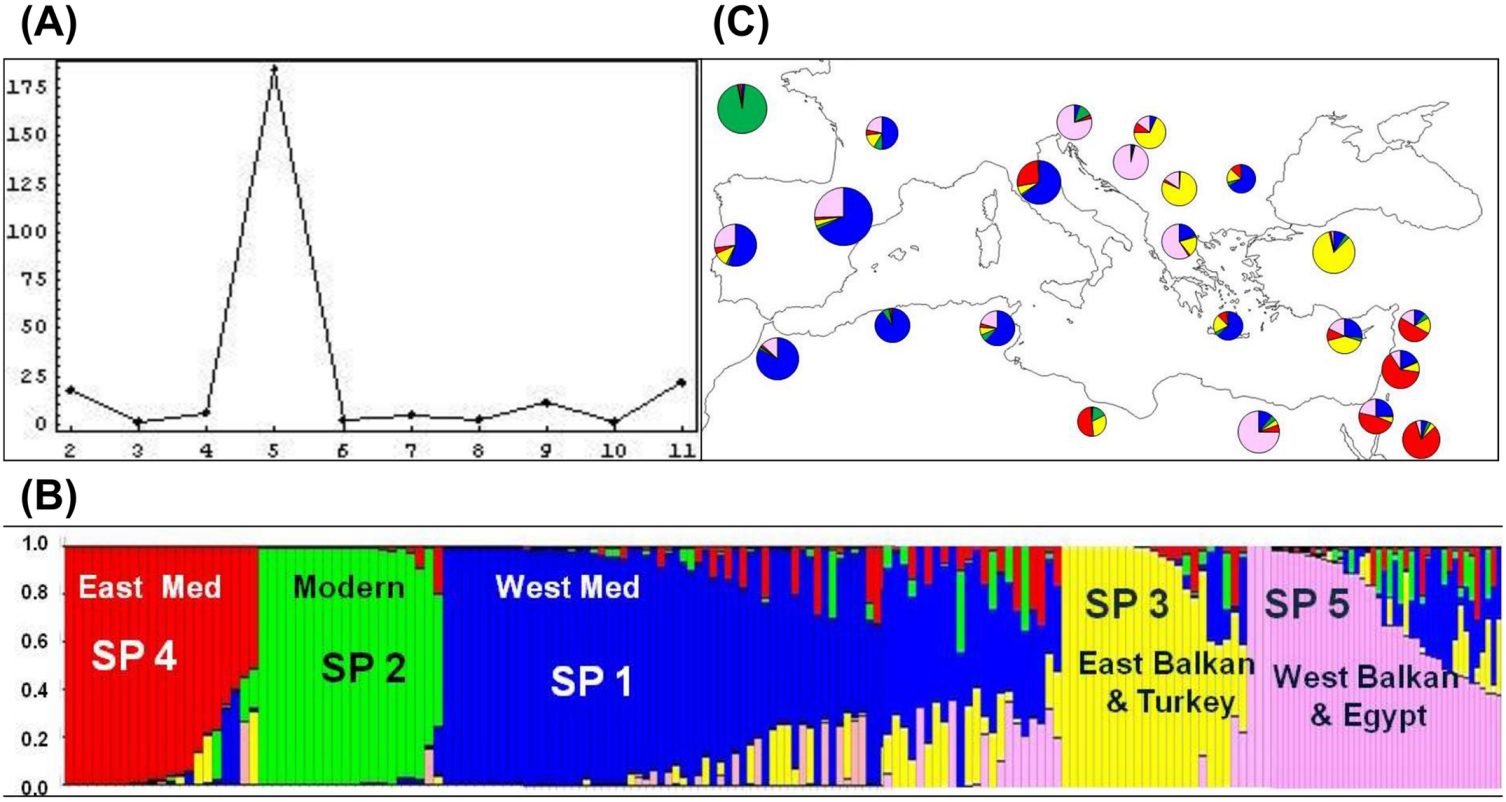
Analysis of the genetic structure of the population. (A) Estimation of the number of subpopulations (SP) according to Evanno’s test [35]. (B) Inferred structure of the durum wheat collection based on 192 genotypes. Each individual is represented by a coloured bar with length proportional to the estimated membership to each of the five subpopulations. (C) Geographic distribution of the durum wheat subpopulations within the Mediterranean Basin. The size of circles is proportional to the number of accessions from each geographic origin.

The analysis conducted using the STRUCTURE software [34] showed that 172 of the 192 accessions could be grouped into five subpopulations ranging from 20 to 73 members each when the estimate of lnPr(X/K) reached a minimum stable value [35] (Fig 1a and Table 1). The inferred population structure for K = 5 showed that 67% of the accessions have a membership coefficient (*qi*) to one of the subpopulations higher than 0.8, while the rest could be considered as admixed (*qi*≤0.8). Nineteen accessions (9.9%) were not included in any of the subpopulations. Within each subpopulation the percentage of accessions with *qi*>0.8 ranged from 57% for subpopulation 1 to 95% for subpopulation 2, the last including only modern accessions. According to the frequency on each subpopultion of accessions collected in a given country (Fig 1b), the subpopulations could be classified according to their geographic origin (Fig 1c). Following this criterion, subpopulation 1 included 73 wheat accessions, mainly from the west area of the Mediterranean Basin (87%), and 13% of accessions from the eastern Mediterranean Basin and the Balkan Peninsula. Subpopulation 2 grouped the whole set of modern cultivars. Subpopulation 3 clustered 21 accessions from Turkey, Cyprus and the eastern Balkan Peninsula. Seventeen cultivars from the eastern Mediterranean Basin were represented in subpopulation 4. Additionally this subpopulation included the Italian cultivars ‘IG-83920’, ‘Hymera’, ‘Aziziah 17/45’ and ‘Capeiti’. Finally, subpopulation 5 was represented by 25 accessions from the western Balkans and Egypt, but it also included 7 Spanish, 4 Portuguese, 1 Moroccan and 1 Tunisian LR.

The 44 selected SSR markers were highly polymorphic, identifying 448 alleles in the 192 durum wheat accessions. The number of alleles per locus ranged between 4 and 21, with a mean of 10 alleles per locus (Table 1). The allelic frequencies (*p*) ranged from 0.003 to 0.857, with a mean of 0.098. A total of 226 alleles might be considered rare as they have a *p*<0.05. The mean genetic diversity values estimated were *Ho* = 0.14 and *He* = 0.71. Wright’s fixation index (*F*), which compares *He* with *Ho* to estimate the degree of allelic fixation, ranged from -0.65 for wms601 to 0.99 for wmc486, with a mean value of 0.79 for the whole set of cultivars. Taking into account the mean values for the different subpopulations, modern cultivars showed lower heterozygosity and higher fixation index mean values, with 59% of the markers (26) having all the alleles fixed (*Ho* = 0 and *F* = 1) (Table 1).

The five durum wheat subpopulations showed a total genetic diversity (*H*_*T*_) ranging from 0.4835 for the modern cultivars (subpopulation 2) to 0.6979 for subpopulation 5, including western Balkan and Egyptian accessions (Table 2). The high value for the genetic diversity among all the accessions (*H*_*T*_ = 0.7080) and the lower value for the genetic diversity among subpopulations (*D*_*ST*_ = 0.1225) resulted in a genetic differentiation value (*G*_*ST*_) of 0.1730, indicating that genetic variation was relatively low between subpopulations (only 17.3% of the variability), while most of the diversity lies within the subpopulations (82.7%).

**Table 2:** Genetic diversity of the five subpopulations (SP).

	Genotypes	*H*_*T*_	*H*_*S*_	*D*_*ST*_	*G*_*ST*_
**Total**	192	0.7080	0.5855	0.1225	0.1730
**SP1—West Mediterranean**	73	0.6523			
**SP2—Modern cultivars**	20	0.4835			
**SP3—East Balkan & Turkey**	21	0.5824			
**SP4—East Mediterranean**	21	0.5112			
**SP5—West Balkan & Egypt**	38	0.6979			

Total genetic diversity (*H*_*T*_), genetic diversity within subpopulations (*H*_*S*_), genetic diversity between subpopulations (*D*_*ST*_) and coefficient of genetic differentiation (*G*_*ST*_) calculated from SSR data according to the STRUCTURE analysis.

**Fig 2:**
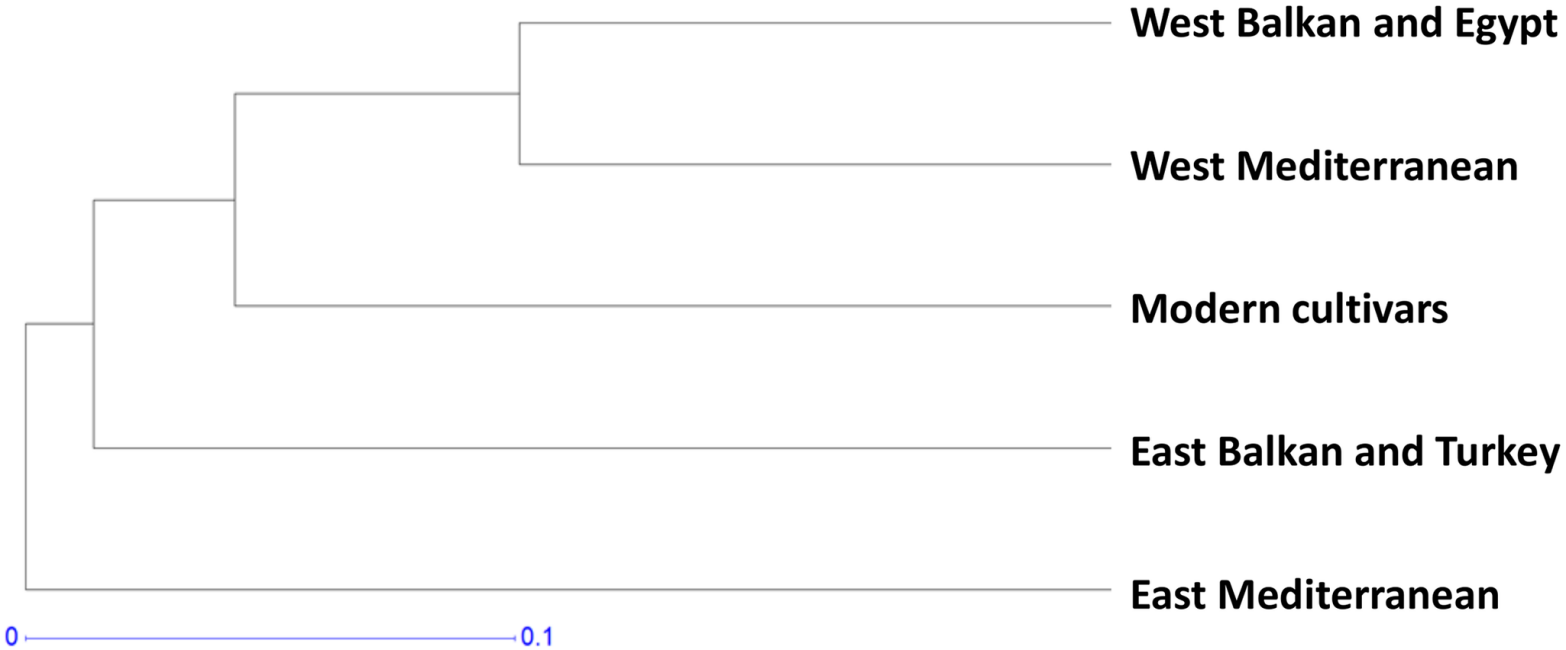
Genetic distance between subpopulations. UPGMA dendrogram based on Nei (1972) genetic distances between the Mediterranean durum wheat subpopulations defined by STRUCTURE analysis.

An UPGMA cluster was obtained from the genetic distance matrix [37] (S3 Table) of the five subpopulations (Fig 2). The genetic distance matrix revealed that the least genetic distance existed between subpopulation 1 and subpopulation 5 (0.2489) and the greatest (0.4886) between subpopulation 3 and subpopulation 4. The dendrogram distributed the subpopulations into three groups, with subpopulation 4 showing the maximum genetic distance and separated in a distinct group.

The molecular variance factor obtained for the five subpopulations from analysis of the molecular variance (AMOVA) was compared as a further measure of the genetic diversity within the durum wheat accessions. The results of AMOVA indicated that most of the genetic variation (69%) between the 172 accessions structured in 5 subpopulations could be explained by variation within subpopulations, while the variation between them was 13%. Finally, variation within accessions represented 18% of the total variance (Table 3).

**Table 3:** Analysis of molecular variance (AMOVA).

Source	df	SS	MS	Estimated variance	Variance (%)
Between subpopulations	4	636	159	2.1	13
Within subpopulations	168	4364	26.0	11.5	69
Within genotypes	172	516	2.99	3.0	18

Analysis of molecular variance between accessions based on population structure.

### Phenotypic data

The ANOVA of phenotypic data revealed that except for yield and TKW, the site effect was more important in the phenotypic expression of traits than the year effect (Table 4). The environment (combination of year and site) accounted for between 8.7% (for PH) and 81.1% (for days at booting) of total variation. The genotype effect explained the largest variation for PH, NGm^2^ and yield, while it accounted for less than 10% of total variation for CDW in all growth stages. The partitioning of the sum of squares of the genotype effect into differences between and within subpopulations indicated that differences between subpopulations were significant for all traits except CDW, and that statistically significant variation existed within subpopulations for number of grains per m^2^ and PH. In general, interaction effects accounted for a low percentage of total variance.

**Table 4:** Analysis of variance.

Source of Variation	df	Yield	NSm^2^	NGm^2^	TKW	HI	CDW_21_	CDW_33_	CDW_65_	CDW_87_	PH	Days_45_	Days_55_	Days_65_	Days_87_
Year	2	19.0***	3.8***	2.7***	48.1***	2.5***	11.3***	12.5***	10.4***	7.9***	0.2***	0.2***	0.4***	2.4***	19.4***
Site	1	8.3***	16.6***	7.6***	0.7***	23.3***	55.2***	49.5***	40.1***	66.9***	8.5***	79.9***	73.7***	66.9***	55.7***
Genotype	191	28.3***	18.1***	37.5***	22.5***	15.8***	4.31	3.1	8.1***	4.0*	78.2***	12.1***	17.2***	20.5***	9.0***
Between SP	4	54.3***	15.0***	55.9***	19.4***	49.0***			8.8	10.7	58.8***	49.5***	50.2***	48.5***	35.3***
Within SP	168	38.0	68.1	37.2*	73.0	47.4			81.7	84.0	31.3***	38.6	37.1	39.6	47.9
Year x Site	2	4.1***	18.9***	9.4***	14.2***	27.4***	27.4***	19.3***	11.5***	4.7***	3.3***	1.5**	1.9***	1.6***	9.2***
Year x Genotype	382	11.6	15.1	12.9	5.6	15.0*	15.0*	6.3	10.8	6.2	3.1	2.0**	1.5*	2.1	2.3
Site x Genotype	191	10.9	13.8***	10.0	4.2***	4.4	4.4	3.1	8.3***	4.1*	3.4	2.9***	4.0***	4.6***	2.4***

Percentage of the sum of squares (SS) of the ANOVA for the 14 phenotypic traits of 192 durum wheat genotypes grown in six environments. The 19 unclassified accessions were not included in the partitioning of the genotype effect. NSm^2^, number of spikes per m^2^; NGm^2^, number of grains per m^2^; TKW, thousand kernel weight; HI, harvest index; CDW, crop dry weight; Days, number of days from sowing. Subscripts refer to growth stages according to the Zadoks scale [30].

* *P*<0.05,

** *P*<0.01,

*** *P*<0.001.

Mean values of phenotypic traits across environments for the five subpopulations are shown in Table 5. Mean yield of subpopulations ranged from 2.6 t ha^-1^ in subpopulation 5 to 4.0 t ha^-1^ in subpopulation 2, with the remaining three subpopulations showing similar values of 3.1 t ha^-1^. Modern cultivars had the highest NGm^2^ and HI and the shortest cycle length. For landraces, the highest NSm^2^ were recorded in subpopulations 3 and 4 and the lowest in subpopulation 1. The NGm^2^ was highest in subpopulation 4. Landraces from the eastern Balkan Peninsula and Turkey (subpopulation 3) and the western Mediterranean (subpopulation 1) had the heaviest grains, whereas those from the western Balkans and Egypt (subpopulation 5) and the eastern Mediterranean (subpopulation 4) had the lightest (Table 5). The latter subpopulations also showed the lowest HI and the shortest plants. Landraces from the eastern Mediterranean Basin were the earliest and those including Balkan accessions had the longest cycle length.

**Table 5:** Mean comparisons.

SP	1	2	3	4	5
Geographic area Trait	West Mediterranean	Modern cultivars	East Balkan & Turkey	East Mediterranean	West Balkan & Egypt
Yield (t ha^-1^)	3.1±0.3^b^	4.0 ± 0.3^a^	3.1±0.2^b^	3.1±0.3^b^	2.6±0.3^c^
Number of spikes per m^2^	398±39^c^	446 ± 29^a^	434±34^ab^	437±34^ab^	419±54^b^
Number of grains per m^2^	6461±827^c^	8999±730^a^	6319±444^cd^	7088±662^b^	5988±849^d^
Thousand Kernel Weight (g)	48.5±4.6^a^	44.7±3.3^b^	48.8±2.4^a^	44.3±2.3^b^	44.0±5.0^b^
Harvest Index	0.37±0.1^c^	0.43±0.1^a^	0.37±0.1^c^	0.39±0.1^b^	0.36±0.1^c^
Crop Dry Weight _21_ (g m^-2^)	213±25^a^	213±20^a^	215±24^a^	217±27^a^	215±20^a^
Crop Dry Weight _33_ (g m^-2^)	715±76^a^	745±67^a^	739±99^a^	709±79^a^	707±64^a^
Crop Dry Weight _65_ (g m^-2^)	1351±122^a^	1324±123^a^	1378±119^a^	1221±110^a^	1334±155^a^
Crop Dry Weight _87_ (g m^-2^)	1806±127^a^	1842±144^a^	1813±105^a^	1745±105^a^	1709±128^a^
Plant Height (cm)	126±9^a^	85±4^c^	130±6^a^	108±6^b^	127±15^a^
Days sowing—booting	143±2^b^	138±2^c^	145±2^a^	139±2^c^	145±3^a^
Days sowing—heading	152±2^a^	145±2^b^	153±2^a^	146±2^b^	153±3^a^
Days sowing—anthesis	160±2^b^	154±2^c^	161±2^a^	155±2^c^	161±3^a^
Days sowing—maturity	191±2^a^	187±2^b^	191±2^a^	188±2^b^	192±3^a^

Mean values ± SD of phenotypic traits for the five subpopulations. Data are means across six environments. Means within files with the same letter are not significantly different at *P*<0.05 according to Duncan’s test.

### Relationship between genetic and phenotypic structures

**Fig 3:**
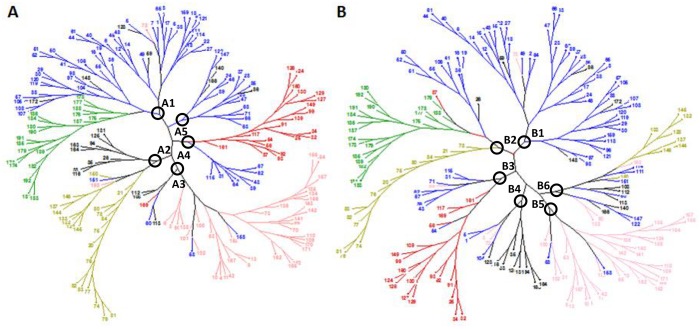
Un-rooted neighbour-joining dendrograms. (A) A simple matching dissimilarity matrix calculated from the dataset of 44 SSR data. (B) Euclidean distances calculated with the standardized mean phenotypic data across environments. Colours of branches correspond with the five subpopulations obtained by STRUCTURE analysis according to Fig 1. Numbers correspond to entries in Table 1.

Cluster analyses were performed based on SSR markers and phenotypic data (Fig 3). The dendrogram generated with SSR data has five major clusters that are mainly in agreement with the five subpopulations given by STRUCTURE (Fig 3A). Cluster A1 included most of the western Mediterranean cultivars (subpopulation 1) together with the modern cultivars (subpopulation 2). The cluster includes one Portuguese cultivar from subpopulation 5 (‘Alentejo’) that can be considered admixed from subpopulation 1 (*q*_1_ = 0.34) and subpopulation 5 (*q*_5_ = 0.50), and four cultivars that were not assigned to any subpopulation, from Portugal, Cyprus, Morocco and Tunisia. Cluster A2 was divided into two branches, the first one including nine cultivars not assigned to any subpopulation, all of them from the eastern Mediterranean Basin, and the second one grouping the cultivars from the eastern Balkans and Turkey (subpopulation 3). This branch included the Greek cultivar ‘Rapsani’ (subpopulation 1, *q*_1_ = 0.56; *q*_3_ = 0.32) and the Egyptian cultivar ‘31’ (subpopulation 5, *q*_5_ = 0.64; *q*_3_ = 0.27). Cluster A3 corresponds with subpopulation 5 (western Balkans and Egypt). The main group of the cluster included all but two of the accessions belonging to this subpopulation and two cultivars from subpopulation 1, the Italian cultivar ‘Balilla Falso’ (*q*_1_ = 0.72) and the French cultivar ‘Rubio enlargado d’Atlemteje’ (*q*_1_ = 0.71). A second group within the cluster included five cultivars, one from subpopulation 1, another from subpopulation 4 and three unstructured. Cluster A4 is divided into two branches: the first grouped cultivars from the western Mediterranean Basin (subpopulation 1) and the second grouped all but one accession from subpopulation 4 (eastern Mediterranean). Finally, cluster A5 included the rest of subpopulation 1 together with three unstructured cultivars.

Cluster analysis using the mean values of the 14 phenotypic traits resulted in an un-rooted tree with 6 main clusters (Fig 3B). As reported previously for SSR data, most of the cultivars were grouped in clusters corresponding to the classification defined by STRUCTURE. Cluster B1 grouped most of the western Mediterranean cultivars, including three Portuguese accessions not included in subpopulation 1: ‘Caxudo de sete espigas’ (subpopulation 5, *q*_1_ = 0.36; *q*_5_ = 0.63) and the unstructured accessions ‘Marques’ and ‘Lobeiro de grao escuro’. Cluster B2 was divided into three branches, each representing a different subpopulation. The first branch clustered accessions from subpopulation 1 (western Mediterranean); the second branch the modern cultivars (subpopulation 2), including the Italian accession ‘Capeiti’; and the third branch subpopulation 3 (eastern Balkans and Turkey). Cluster B3 basically corresponds to the fourth cluster obtained using SSR data with the inclusion of the Portuguese cultivar ‘Dezassete’ (subpopulation 5, *q*_1_ = 0.41; *q*_5_ = 0.50) within the subpopulation 1 accessions. Cluster B4 was divided into two groups: the first one a mix of accessions from subpopulation 1 (3) and subpopulation 5 (2) from the western Mediterranean Basin and one unstructured accession; and the second one eight unstructured accessions corresponding to one of the branches of cluster A2 in the SSR dendrogram. Cluster B5 included most of the subpopulation 5 cultivars, including two subpopulation 1 accessions, as reported above for the main group of cluster A3 for molecular data. Finally, cluster B6 grouped the rest of subpopulation 1, 3, 5 and unstructured accessions.

## Discussion

### Genetic structure and diversity

The analysis of the population structure showed a noticeable division into landraces and modern cultivars and a clear classification of landraces according to their geographical origin. Excluding the modern cultivars, the Bayesian-based analysis without a priori assignment of accessions to population classified 152 landraces into four subpopulationscategorized according to their geographical origin. Accessions showed a strong structure with an eastern-western geographical pattern formed by four clearly defined groups: eastern Mediterranean (subpopulation 4), Eastern Balkans and Turkey (subpopulation 3), western Balkans and Egypt (subpopulation 5) and western Mediterranean (subpopulation 1). This structure agrees with the pattern of dispersal of wheat from east to west in the Mediterranean Basin [1]. Therefore, the low genetic distance estimated between subpopulations 1 and 5 may be explained by geographic proximity. However, this explanation is not valid for elucidating the distance between subpopulations 3 and 4, which were close geographically but showed the largest genetic distance. This finding, jointly with the splitting of landraces from the Balkan Peninsula into two different genetic subpopulations, supports the hypothesis of two different origins of the Balkan durum wheats, as suggested by previous results [4, 41]. Moreover, Dedkova et al. [42] demonstrated that *T*. *dicoccum* accessions from the former Yugoslavia, Bulgaria and Russia do not carry the 7A:6B translocation that is common in the *dicoccum* accessions from western Mediterranean countries; they proposed a division of European *T*. *dicoccum* into two groups: western European and Volga-Balkan. In agreement with this theory, the results of the population structure obtained in the current study show that landraces of subpopulation 3 may have a different origin than those of subpopulation 5. The largest genetic distance between subpopulation s 3 and 4 than between subpopulations 4 and 5 allows us to hypothesize that subpopulation 3 may be the one including landraces from the Volga region.

The results of the present study showed the suitability of the groups resulting from the SSR marker analysis for depicting the genetic structure and diversity of durum wheat landraces across the Mediterranean Basin. Moreover, the population structure ascertained in this study may be very useful for improving the reliability of future association-mapping studies. It is well known that population structure influences linkage disequilibrium due to the presence of population stratification and an unequal distribution of alleles within groups, which can result in spurious associations.

The subpopulations showed a membership coefficient of the accessions higher than 0.8 in a range from 57% to 76%, suggesting the presence of admixture. The admixture in tetraploid Mediterranean wheat accessions could result from the incorporation in landraces of alleles from more than a single gene pool due to the spread of wheat from more than a single ancestral population, as has been suggested [18]. An alternative reason could be that the gene flow between different cultivars may have occurred in the past through the introduction of new genotypes into fields. The exchange of germplasm between different Mediterranean regions due to the expansion of the Arabian Empire during the Middle Ages has been suggested as a possible cause of admixture [13].

Genetic diversity in wheat was increasingly narrowed down during the second part of the 20^th^ century due to the wide adoption of improved semi-dwarf wheat cultivars. A number of studies consider collections of wheat landraces as sources of putatively lost variability that are able to provide new favourable genes/alleles to be introgressed into modern cultivars [3 and references therein]. Although Mediterranean landraces have been shown to be particularly valuable due to their huge genetic diversity and the presence of accessions with high resilience to abiotic stresses, resistance to pests and diseases and high grain quality [3, 4], the large genetic distance estimated between modern cultivars and all landrace subpopulations shows the low use of durum landraces by durum wheat breeding programmes.

Exploiting the variability of wheat landraces requires previous knowledge of their genetic diversity. In this study, we used 44 SSR markers to quantify the genetic diversity existing in a set of 172 durum wheat landraces from the Mediterranean Basin and 20 modern cultivars. The number of alleles identified in this study, and the value estimated for the genetic diversity of the collection (0.71), were higher than the values reported by previous studies involving durum wheat collections (*He* values between 0.55 and 0.68) [43–47], and also than those found in bread wheat collections (*He* values between 0.54 and 0.63 [15–17, 20, 48]. The high level of genetic diversity found in this study may be due to the presence of many unique alleles in landraces from different areas of the region, so it is essential to assess the genetic structure of the population.

The coefficient of gene differentiation (*G*_*ST*_) is directly proportional to the amount of variation among populations [49]. As a consequence of the low value of the genetic diversity between subpopulations(*D*_*ST*_) obtained in the current study, *G*_*ST*_ was also low, showing that only 17% of the variability was due to differences between subpopulations, while the remainder was a consequence of the genotypic variability within each subpopulation. The results of the analysis of molecular variance were in agreement with the low value of *G*_*ST*_, as only 13% of the variation in the durum wheat accessions was due to variation between subpopulations. These results indicate that, though landraces of different geographic origin were polymorphic enough to trace a consistent geographical pattern, the genetic variability within the set of genotypes of a common origin was much wider. The presence of a large number of unique putative alleles in this collection, which have already been identified for glutenin subunits [9], may significantly contribute to this large variability.

As expected, gene diversity was the lowest for the modern cultivars due to the selection pressure applied by breeders in the last few decades. Several authors [1, 50] have postulated that durum wheat spread across the Mediterranean Basin from the Fertile Crescent (10,000 BP) via Turkey (8500 BP), the Balkan Peninsula, Greece and Italy (8000 BP), and from there to North Africa and the Iberian Peninsula (7000 BP). Within the landrace subpopulations, gene diversity was lower in the eastern Mediterranean group, indicating that the diversity of wheat increased during its dispersal from its area of domestication to the western Mediterranean Basin. According to these results, Ren et al. [51] using a worldwide collection of durum wheat found that the Middle East region showed moderate levels of genetic diversity, lower than those from South America, North America, and Western Europe. Authors concluded that the centres of diversity were not confined exclusively to their centres of origin. More recently in a study of Ethiopian landraces [52] authors found higher level of diversity than in a set of Mediterranean landraces, suggesting that the evolutionary history of wheat in East Africa is different.

### Agronomic performance

The ANOVA showed the large effect of environmental conditions on the phenotypic expression of agronomic traits. The environmental conditions during the three years of field experiments were typical of a Mediterranean climate with a pattern of increasing temperatures during the spring and uneven distribution of rainfalls [25]. The sum of the environmental effects (year and site) accounted for a larger variation than the genotype for most traits, particularly the number of days to different phenological stages (from 69% to 81% depending on the growth stage) and CDW (from 50% to 75%). Modern cultivars had a shorter cycle length than the landraces, a finding that has been explained in previous studies by the introduction of dwarfing genes [53]. Landraces from the Balkan Peninsula (subpopulation 3and 5) took 4 to 7 days more from sowing to reach the different growth stages than those from the eastern Mediterranean Basin, which showed the shortest periods to any phenological stage among the landraces. The cooler climate of the Balkan Peninsula may have resulted in a lengthening of the growth cycle of the landraces that originated in this area [25]. On the other hand, the high temperatures and low rainfall of the southeastern Mediterranean Basin may have reduced time to heading [25, 54] as an adaptive physiological mechanism for terminal drought escape.

Genotypic differences in CDW were only significant at anthesis, but variability was not found between or within subpopulations. The low variability for biomass in durum wheat has been reported in previous studies involving semi-dwarf durums [55]. In addition, the lack of statistically significant differences for CDW between subpopulation s at physiological maturity and the superior HI of modern cultivars suggest that the plant weight of the landraces compensated for the higher weight allocated in the grains of modern cultivars, leading to similar CDW at maturity. Most of the phenotypic variability in PH was explained by the genotype effect, in agreement with the high heritability of this trait [56], previously associated with the presence of the dwarfing gene *Rht-B1b* [57]. A similar result was reported using a collection of 191 durum wheat elite accessions [14].

The genotype effect explained 16% to 37% of total variation for yield, yield components and HI, while differences between subpopulation accounted for 15% to 56% of variation. Variability within subpopulations was only significant for the number of grains. The high number of spikes recorded in landraces from the eastern Mediterranean Basin was in agreement with the findings of previous studies, which demonstrated that durum wheat yield under warm and dry environments is determined mostly by the number of spikes per unit area, whereas kernel weight predominantly influences grain production in colder and wetter environments [2, 58, 59].

As expected, the HI of Mediterranean landraces was lower than that reported for modern semi-dwarf cultivars. Among the landrace subpopulations, the highest HI was found within the eastern Mediterranean landraces. Moragues [2], using a collection of 52 durum wheat landraces classified according to the dispersal of durum wheat across the Mediterranean Basin (northern and southern dispersal), showed that HI was higher within the southern landraces coming from dryer and warmer areas. These authors suggested that the southern landraces probably had a higher capacity to allocate biomass into grains and a better ability to set grains under stress, which is in agreement with the large NGm^2^ recorded in subpopulation 4. The greater HI of eastern Mediterranean accessions found in this study may also indicate that they were more efficient in using water during the later stages of development than landraces from cooler areas, which is a sign of adaptation to drought environments.

### Relationship between genetic structure and phenotypic performance

Classification of the accessions using the neighbour-joining clustering method based on SSR and phenotypic dissimilarities showed an evident correspondence with results obtained in the analysis carried out using the STRUCTURE software. The clearest case was that of the 20 modern cultivars that were always grouped together. The clustering of the Italian cultivar ‘Capeiti’ (subpopulation 4) jointly with modern cultivars (subpopulation 2) in the phenotypic tree may be due to its extensive use by Italian breeders as a hallmark founder for the development of drought-tolerant cultivars [60].

The clustering of accessions in dendrograms based on SSR and phenotypic data were essentially coincident. However, in cases of accessions with large admixture, the dendrograms concurred in locating them in branches corresponding to different subpopulations than those assigned by STRUCTURE. This was the case of the Greek LR ‘Rapsani’ and the Egyptian LR ‘31’. Interestingly, 5 of the 19 accessions not assigned to any subpopulations by STRUCTURE were located in dendrograms in branches close to accessions of similar geographic origin. This was the case of the landraces ‘Marques’ and ‘Lobeiro de grao escuro’ from Portugal, ‘Hamira’ from Tunisia, and ‘Haj Mouline’ from Morocco, which were all located jointly with western Mediterranean accessions in both dendrograms.

In addition, some of admixed cultivars from STRUCTURE that originated in Israel, Jordan, Lebanon and Syria were placed close to the cluster defined by DARWin with molecular data containing accessions from the eastern Balkans and Turkey (subpopulation 3).

Accessions included in subpopulation3 (eastern Balkans and Turkey) by STRUCTURE were separated into two different branches in the dendrogram drawn with phenotypic data. One of them contained the accessions from Cyprus and Turkey ‘Vroulos’, ‘IG-82549’, ‘BGE-018192’, ‘BGE-018353’, ‘BGE-019262’ and ‘BGE-019264’, and the other clustered all Serbian and Macedonian landraces included in subpopulation 3. This result not only supports the hypothesis that subpopulation 3 contains accessions from the Volga region, but identifies them on the basis of their agronomic performance.

A previous study [14] using a collection of 191 durum accessions and mainly semi-dwarf elite materials, and including a limited number of founders, found a weak relationship between molecular clustering and phenotypic structure. Only accessions closely related to the CIMMYT hallmark founder ‘Altar 84’, the ICARDA accessions adapted to continental-dryland areas and the landraces were clustered in both the genetic dissimilarities tree and the tree obtained using Euclidean distances based on standardized phenotypic data across environments. Comparing that study with the current one, it seems that the similarities between genetic distances and adaptive responses is more accurate for landraces than for modern cultivars, likely due to the lack of genetic improvement of the landraces and the incorporation in modern cultivars of genes associated with traits different from those related to adaptive response of genotypes.

## Conclusions

The current study aimed to understand the genetic, phenotypic and geographic structures of a collection of Mediterranean durum wheat cultivars and the relationships between them. The results demonstrated the usefulness of the methodologies used to determine the structure of Mediterranean durums. Landraces and modern cultivars were split into two different groups using either molecular or phenotypic data. Based on data from 44 SSR markers, STRUCTURE software assigned 90% of the accessions to five subpopulations, with the four ones having landraces showing a geographical structure. Unexpectedly, the genetic diversity was greater within subpopulations than between them, which denotes the large variability existing in landraces of a common geographic origin. The study identified a large number of alleles (448), about half of which had a very low frequency and were therefore rare alleles, and an average number of 10 alleles per locus. Gene diversity increased from the eastern to the western Mediterranean Basin, in agreement with the dispersal pattern of wheat from its domestication area.

The un-rooted neighbour-joining dendrogram based on SSR data coincided in essence with the clusters obtained by STRUCTURE, but complemented the information provided by it when accessions showed large admixture. The strong coincidence between dendrograms based on molecular and phenotypic data indicates i) the suitability of the phenotypic traits used in the current study for differentiating groups of accessions with similar field performance, and ii) the robust relationship between the phenotypic expression of traits and the genetic background underlying them. Among the subpopulation that included durums from the eastern Balkans and Turkey, the assessment of phenotypic traits based on yield, yield components and crop phenology was also useful for separating those from Macedonia and Serbia from those from Turkey, Cyprus and Greece, which very likely had a different origin.

This is the first study using durum wheat Mediterranean landraces and modern cultivars that shows a reliable relationship between genetic and phenotypic population structures, and the connection of both with the geographic origin of landraces. The results of the current study demonstrate that, when appropriate markers in number and distribution are used and phenotyping is adequately conducted, high similarities may be found between genetic distances and the adaptive response of durum wheat.

## Supporting Information

S1 Table Durum wheat cultivars. Durum wheats included in the study. Entries from 001 to 172 correspond to landraces and those from 173 to 192 correspond to modern cultivars.
(DOCX)

S2 Table Experimental details of the six field experiments. (DOCX)

S3 Table Genetic distances. Genetic distance based on [37] between the subpopulations identified by STRUCTURE analysis.
(DOCX)
