# Supplementary material for: Genetic Structure of Modern Durum Wheat Cultivars and Mediterranean Landraces Matches with Their Agronomic Performance
Source: PLoS One. 2016 Aug 11;11(8):e0160983. doi: 10.1371/journal.pone.0160983 (PMC4981446; doi:10.1371/journal.pone.0160983)
Supplement: S1 Table — Durum wheats included in the study. Entries from 001 to 172 correspond to landraces and those from 173 to 192 correspond to modern cultivars. (DOCX) [file pone.0160983.s001.docx]

**S1 Table. Durum wheat cultivars.**

| Entry | Cultivar^a^ | Country | SP^b^ | Entry | Cultivar | Country | SP |
| --- | --- | --- | --- | --- | --- | --- | --- |
| 027 | IG-92895 | Algeria | 1 | 132 | 248-VII/7 | Macedonia | 3 |
| 028 | IG-92967 | Algeria | 1 | 133 | 259-VII/12 | Macedonia | 3 |
| 029 | IG-93030 | Algeria | 1 | 137 | VII/13-X11 | Macedonia | 3 |
| 030 | IG-93621 | Algeria | 1 | 144 | 196/71 | Macedonia | 3 |
| 031 | IG-94009 | Algeria | 1 | 146 | II/4 | Macedonia | 3 |
| 089 | Dur de Medeah | Algeria | 1 | 128 | Belgrade 9 | Serbia | 3 |
| 018 | Tchirpan | Bulgaria | 1 | 145 | 1575 | Serbia | 3 |
| 019 | Lozen 76 | Bulgaria | 1 | 074 | BGE-018192 | Turkey | 3 |
| 036 | IG-96802 | Creta | 1 | 075 | BGE-018350 | Turkey | 3 |
| 037 | IG-96851 | Creta | 1 | 076 | BGE-018351 | Turkey | 3 |
| 122 | Muri | Cyprus | 1 | 077 | BGE-018353 | Turkey | 3 |
| 062 | Reading | Egypt | 1 | 078 | BGE-018354 | Turkey | 3 |
| 111 | Beladi Rouge | France | 1 | 079 | BGE-019262 | Turkey | 3 |
| 114 | Tounse | France | 1 | 080 | BGE-019263 | Turkey | 3 |
| 115 | Trigo Glutinoso | France | 1 | 081 | BGE-019264 | Turkey | 3 |
| 158 | R. enlargado d’Atlemteje | France | 1 | 082 | BGE-019265 | Turkey | 3 |
| 151 | Rapsani | Greece | 1 | 083 | BGE-019266 | Turkey | 3 |
| 022 | Carlantino | Italy | 1 | 084 | BGE-019270 | Turkey | 3 |
| 023 | Cicirelo | Italy | 1 | 169 | Sinai No.8 | Egypt | 4 |
| 024 | IG-83905 | Italy | 1 | 130 | Etith | Israel | 4 |
| 051 | Carlo jucci | Italy | 1 | 160 | Hati | Israel | 4 |
| 052 | Senatore Cappelli | Italy | 1 | 025 | IG-83920 | Italy | 4 |
| 055 | Trinakria | Italy | 1 | 054 | Hymera | Italy | 4 |
| 057 | Razza 208 | Italy | 1 | 056 | Aziziah 17/45 | Italy | 4 |
| 058 | Balilla Falso | Italy | 1 | 087 | Capeiti | Italy | 4 |
| 064 | Milazzo | Italy | 1 | 099 | Safra Jerash | Jordan | 4 |
| 066 | Razza 181 | Italy | 1 | 124 | Harani Auttma | Jordan | 4 |
| 088 | Razza 96 | Italy | 1 | 126 | Horani Howawi | Jordan | 4 |
| 159 | Reyati | Lebanon | 1 | 127 | Zugbieh Sutra | Jordan | 4 |
| 096 | Maghoussa | Morocco | 1 | 129 | Zoghbiyeh Safra | Jordan | 4 |
| 097 | Merzaga | Morocco | 1 | 139 | Safra Maan | Jordan | 4 |
| 098 | Red Beard | Morocco | 1 | 149 | 26 | Jordan | 4 |
| 107 | Morocco | Morocco | 1 | 091 | 9923 | Lebanon | 4 |
| 108 | Saffi | Morocco | 1 | 092 | 9929 | Lebanon | 4 |
| 118 | Ble Dur 250 | Morocco | 1 | 093 | 9935 | Lebanon | 4 |
| 119 | Oned Zenati | Morocco | 1 | 117 | Hourah | Lebanon | 4 |
| 120 | Mahmoudi C | Morocco | 1 | 161 | Tripshiro | Libya | 4 |
| 121 | Maghoussa Amizmiz | Morocco | 1 | 032 | IG-95812 | Syria | 4 |
| 147 | Cobros | Morocco | 1 | 034 | IG-95847 | Syria | 4 |
| 061 | Raposinho | Portugal | 1 | 100 | Dalmatia 1 | Croatia | 5 |
| 063 | Durazio Rijo | Portugal | 1 | 101 | Dalmatia 3 | Croatia | 5 |
| 065 | Raspinegro | Portugal | 1 | 135 | 440-IX/96 | Croatia | 5 |
| 067 | Anafil | Portugal | 1 | 136 | 441-IX/97 | Croatia | 5 |
| 068 | Espanhol | Portugal | 1 | 157 | 56 | Croatia | 5 |
| 071 | Amarelo Barba Preta | Portugal | 1 | 053 | Milagro | Egypt | 5 |
| 085 | Tremes rijo | Portugal | 1 | 095 | D-2 | Egypt | 5 |
| 001 | Arisnegro de Tenerife | Spain | 1 | 109 | 5P4 | Egypt | 5 |
| 002 | Basto Duro | Spain | 1 | 110 | 1P1 | Egypt | 5 |
| 005 | Candeal de Salamanca | Spain | 1 | 152 | Giza 2 | Egypt | 5 |
| 006 | Colorado de Jerez | Spain | 1 | 162 | 2751 | Egypt | 5 |
| 007 | Enano de Andújar | Spain | 1 | 165 | MG 26429 | Egypt | 5 |
| 008 | Fartó | Spain | 1 | 167 | 28 | Egypt | 5 |
| 012 | Pinet | Spain | 1 | 168 | 31 | Egypt | 5 |
| 014 | Raspinegro Canario | Spain | 1 | 170 | Mishriki | Egypt | 5 |
| 015 | Raspinegro de Alcalá Guadaira | Spain | 1 | 171 | Girgeh | Egypt | 5 |
| 016 | Recio de Almería | Spain | 1 | 141 | FAO 29.946 | Greece | 5 |
| 017 | Verdial | Spain | 1 | 142 | FAO 29.952 | Greece | 5 |
| 038 | Alonso | Spain | 1 | 143 | FAO 29.968 | Greece | 5 |
| 039 | Andalucía 344 | Spain | 1 | 138 | VII/18-X24 | Macedonia | 5 |
| 040 | Azulejo de Villa del Río | Spain | 1 | 134 | 356-I/9 | Montenegro | 5 |
| 041 | Blancal | Spain | 1 | 153 | 23 | Montenegro | 5 |
| 043 | Claro de Balazote | Spain | 1 | 154 | 33 | Montenegro | 5 |
| 044 | Entrelargo de Montijo | Spain | 1 | 155 | 37 | Montenegro | 5 |
| 045 | Farto cañifino | Spain | 1 | 156 | 42 | Montenegro | 5 |
| 046 | Rubio de Miajadas | Spain | 1 | 090 | Zoco Yebel Hebil | Morocco | 5 |
| 047 | Rubio de Montijo | Spain | 1 | 069 | Dezassete | Portugal | 5 |
| 048 | Ruso | Spain | 1 | 070 | Durazio Rijo Glabro | Portugal | 5 |
| 049 | Semental | Spain | 1 | 072 | Alentejo | Portugal | 5 |
| 050 | Recio de Cañete | Spain | 1 | 073 | Caxudo de sete espigas | Portugal | 5 |
| 104 | Souri | Tunisia | 1 | 003 | Blanco de Corella | Spain | 5 |
| 105 | Realforte | Tunisia | 1 | 004 | Blanquillo | Spain | 5 |
| 106 | Biskri | Tunisia | 1 | 009 | Griego de Baleares | Spain | 5 |
| 060 | Mindium | Turkey | 1 | 010 | Gros de Cerdaña | Spain | 5 |
| 173 | Amilcar | Modern | 2 | 011 | Heraldo del Rhin | Spain | 5 |
| 174 | Ancalei | Modern | 2 | 013 | Pisana cañihueca | Spain | 5 |
| 175 | Arment | Modern | 2 | 042 | Blanquillón de Boñar | Spain | 5 |
| 176 | Astigi | Modern | 2 | 102 | 1356-31 | Tunisia | 5 |
| 177 | Boabdil | Modern | 2 | 123 | Akathiotico Naurotheri | Cyprus | na |
| 178 | Bolido | Modern | 2 | 140 | FAO 29.912 | Cyprus | na |
| 179 | Bolo | Modern | 2 | 112 | De Santa Marta | France | na |
| 180 | Claudio | Modern | 2 | 113 | Iumillo | France | na |
| 181 | Gallareta | Modern | 2 | 094 | Abu Fashit | Israel | na |
| 182 | Hispasano | Modern | 2 | 131 | Juljulith | Israel | na |
| 183 | Jupare | Modern | 2 | 163 | JM-3987 | Israel | na |
| 184 | Kronos | Modern | 2 | 164 | JM-3989 | Israel | na |
| 185 | Meridiano | Modern | 2 | 125 | Salti na Zinia | Jordan | na |
| 186 | Ocotillo | Modern | 2 | 026 | IG-84856 | Lebanon | na |
| 187 | Senadur | Modern | 2 | 116 | 9918 | Lebanon | na |
| 188 | Simeto | Modern | 2 | 148 | Haj Mouline | Morocco | na |
| 189 | Sula | Modern | 2 | 059 | Marques | Portugal | na |
| 190 | Svevo | Modern | 2 | 086 | Lobeiro de grao escuro | Portugal | na |
| 191 | Vitron | Modern | 2 | 166 | 18/71 | Serbia | na |
| 192 | Vitronero | Modern | 2 | 033 | IG-95841 | Syria | na |
| 020 | Vroulos | Cyprus | 3 | 035 | IG-95931 | Syria | na |
| 021 | IG-82549 | Cyprus | 3 | 103 | Louri AP 5 | Tunisia | na |
| 150 | Mavraani | Greece | 3 | 172 | Hamira | Tunisia | na |

Durum wheats included in the study. Entries from 001 to 172 correspond to landraces and those from 173 to 192 correspond to modern cultivars.

^a^ BGE-numbers are codes from the Centro de Recursos Fitogenéticos (INIA, Madrid, Spain). IG-numbers are codes from the ICARDA Germplasm Bank. PI-numbers are codes from the USDA Germplasm Bank.

^b^ Accessions assigned to a subpopulation (SP) by STRUCTURE, i.e. with a probability higher than 50% of belonging to any of the 5 genetic subpopulations (SP): 1, western Mediterranean; 2, modern cultivars; 3, eastern Balkans and Turkey; 4, eastern Mediterranean; 5, eastern Balkans and Egypt; na, not assigned.
