# Supplementary material for: Genetic Structure of Modern Durum Wheat Cultivars and Mediterranean Landraces Matches with Their Agronomic Performance
Source: PLoS One. 2016 Aug 11;11(8):e0160983. doi: 10.1371/journal.pone.0160983 (PMC4981446; doi:10.1371/journal.pone.0160983)
Supplement: S2 Table — (DOCX) [file pone.0160983.s002.docx]

**S2 Table. Experimental details of the six field experiments.**

| Environment code  Site | GR07  Granada | GR08  Granada | GR09  Granada | LL07  Lleida | LL08  Lleida | LL09  Lleida |
| --- | --- | --- | --- | --- | --- | --- |
| Year | 2007 | 2008 | 2009 | 2007 | 2008 | 2009 |
| Sowing date (dd/mm/yy) | 14/12/06 | 10/12/07 | 22/12/08 | 21/11/06 | 20/11/07 | 20/11/08 |
| Harvest date (dd/mm/yy) | 29/07/07 | 07/07/08 | 05/07/09 | 02/07/07 | 02/07/08 | 15/07/09 |
| Environmental data from sowing to harvest | | | | | | |
| Water input (mm) | 320 | 345 | 337 | 208 | 258 | 237 |
| Mean of daily minimum temperatures (ºC) | 7.5 | 7.0 | 7.1 | 6.1 | 6.5 | 6.3 |
| Mean of daily maximum temperatures (ºC) | 22.8 | 22.1 | 21.9 | 17.4 | 18.6 | 18.3 |
| Accumulated ET_0_ (mm) | 803 | 673 | 614 | 533 | 755 | 623 |
| Average daily relative air moisture (%) | 55.9 | 58.6 | 60.5 | 73.4 | 88.7 | 70.5 |
| Average daily solar radiation (MJ m^-2^ d^-1^) | 19.4 | 18.4 | 17.8 | 14.5 | 14.9 | 15.9 |
