# Supplementary material for: Genetic Structure of Modern Durum Wheat Cultivars and Mediterranean Landraces Matches with Their Agronomic Performance
Source: PLoS One. 2016 Aug 11;11(8):e0160983. doi: 10.1371/journal.pone.0160983 (PMC4981446; doi:10.1371/journal.pone.0160983)
Supplement: S3 Table — Genetic distance based on [37] between the subpopulations identified by STRUCTURE analysis. (DOCX) [file pone.0160983.s003.docx]

S3 table. Genetic distances (Nei 1972) among the groups defined by STRUCTURE analysis.

|  | SP2 | SP3 | SP4 | SP5 |
| --- | --- | --- | --- | --- |
| SP1 | 0.2994 | 0.3553 | 0.3377 | 0.2489 |
| SP2 |  | 0.4485 | 0.4837 | 0.4311 |
| SP3 |  |  | 0.4886 | 0.4408 |
| SP4 |  |  |  | 0.4230 |
